# An oncolytic adenovirus 11p vector expressing adenovirus death protein in the E1 region showed significant apoptosis and tumour-killing ability in metastatic prostate cells

**DOI:** 10.18632/oncotarget.26754

**Published:** 2019-03-08

**Authors:** Haidong Wu, Ya-Fang Mei

**Affiliations:** ^1^ Department of Clinical Microbiology, Virology, Umeå University, Umeå, Sweden; ^2^ Laboratory Medicine, Clinical Microbiology, Umeå University Hospital, Umeå, Sweden

**Keywords:** oncolytic adenovirus 11p vector, expressing adenovirus death protein, prostate tumour treatment, in vitro and in vivo, apoptosis

## Abstract

The usefulness for cancer therapy of replication-competent adenoviral vectors expressing therapeutic genes from the E3 region has been evaluated, but few reports have described replication-competent adenoviruses with insertions at the E1 region in the full viral genome. We investigated in different prostate cancer cells the oncolytic efficacy of the replication-competent adenovirus 11p vectors expressing adenovirus death (RCAd11pADP) and red fluorescence (RCAd11pRFP) proteins from the upstream E1 region. ADP/RFP gene expression was 2-3 logs higher in PC3 and DU145 cells than in LNCaP and RWPE-1 cells. E1A protein expression in PC3 and DU145 cells was notably increased after infection with the RCAd11pADP or RCAd11pRFP vector compared with the Ad11pwt virus. Toxicity assays revealed 2-5-fold greater oncolytic effects of RCAd11pADP compared to Ad11pwt. Although all three viruses suppressed subcutaneous PC3 tumour growth in nude mice, RCAd11pRFP had greater oncolytic effects than did the Ad11pwt virus, and RCAd11pADP exhibited significant anti-tumour effects via apoptosis in a xenograft model. Interestingly, the apoptosis triggered by RCAd11pADP was markedly enhanced in comparison to that by the vector expressing ADP from E3 region. Taken together, our findings suggest that RCAd11pADP can potentially be used for the treatment of prostate metastases in clinical settings.

## INTRODUCTION

Prostate cancer is the most common cancer and the second leading cause of cancer-related death in men in many western countries [1]. Prostate cancer occurs at a very high frequency in older men over 75 years of age. In addition to surgery, radiotherapy and chemotherapy might improve the patient's status, but most become tolerant to hormone therapy after a few years, resulting in the development of metastases. As the survival of patients with metastatic prostate disease remains very low, new alternative therapeutic methods are needed. Gene therapy has great promise for treating cancer, and the most commonly used vector is based on human adenovirus serotype 5 (Ad5), a virus that causes mild respiratory illness in infants but no disease in immunocompetent adults. Ad5 is one of the best-studied viruses, is genetically stable, does not integrate into the host genome and propagates in cell culture in large amounts [2].

During tumour killing, adenoviral vectors target tumours by replicating in and killing cancer cells. Upon completion of the replication cycle, the infected tumour cell undergoes lysis and releases progeny virions that are capable of infecting neighbouring tumour cells, and repeated cycles of vector replication and cell lysis destroy the tumour. In theory, such vectors should spread through and eliminate a tumour. Most of the commonly used oncolytic adenoviruses are Ad5 vectors that carry the full-length E1 region, limited modifications to E1 open reading frames (ORFs), or a modified E1 promoter. To improve oncolytic efficiency, a therapeutic gene has been inserted downstream of the E1A genes or the E3 region [3, 4]. However, Ad5-based vectors also have some disadvantages. Ad5 results in high seroprevalence among the human population. Ad5 utilizes coxsackie-adenovirus receptor (CAR), which is downregulated in tumour cells, as a cellular receptor [5, 6]. To ensure that Ad5-neutralizating antibodies do not reduce the effects of the vector, other adenovirus serotypes with low seroprevalence in the population and no CAR-binding ability have been selected. Accordingly, we previously used species B adenovirus 11p as the vector to deliver a therapeutic gene to prostate cancer cells. Ad11 binds to CD46 and desmoglein-2 as its primary receptors [7, 8]. Expression of CD46 is increased in tumour cells, and more efficient uptake in the brain was observed for a chimeric Ad5/11F vector [9]. Furthermore, the Ad11 virus more efficiently transduces haematopoietic cells than does the Ad5 virus [10].

In our previous study, an insertion site upstream of the E1 region in the Ad11p genome was successfully defined; a 1.7 k-bp expression cassette was introduced into this site, which did not interrupt viral infection, replication, or propagation [11]. Our replication-competent adenovirus 11p (RCAd11pGFP) vectors were able to achieve native tumour killing in populations of prostate and colon cancer cells both *in vitro* and *in vivo* [12, 13], and we recently demonstrated that RCAd11pe1GFP and RCAd11pe3GFP resulted in 3-, 5- and 7-fold higher numbers of infectious virus particles than did the Ad11p wild-type (WT) virus after heat treatment at 47°C for 5 h. Thus, RCA11pe1GFP and RCAd11p3GFP vectors are more stable than is the WT virus [11].

Adenovirus death protein (ADP), originally derived from adenovirus species C and previously named E3-11.6 K, is synthesized during the very late stage of infection, mediating efficient cell lysis and release of adenovirus particles to infect other cells [14]. The major late promoter (MLP) controls overexpression of the ADP gene from the E3 region of the Ad5 vector, which enhances tumour cell apoptosis but does not affect normal cells [15]. Oneal *et al*. [16] have demonstrated that the Ad5-ADP virus combined with radiotherapy efficiently suppresses prostate tumour growth *in vitro* and *in vivo*. With the exception of species C, the ADP gene is absent in various adenoviruses; however, the function of the ADP gene in species B adenoviruses, such as Ad11p, has not yet been elucidated.

In this study, we constructed a novel replication-competent adenovirus 11p vector (RCAd11pADP) expressing the ADP gene upstream of the E1A region and characterized its transduction efficacy and gene expression, replication, and oncolytic effects on prostate cancer cells *in vitro* and *in vivo*.

## RESULTS

### Characteristics of the RCAd11pADP and RCAd11pRFP vectors

**Figure 1:**
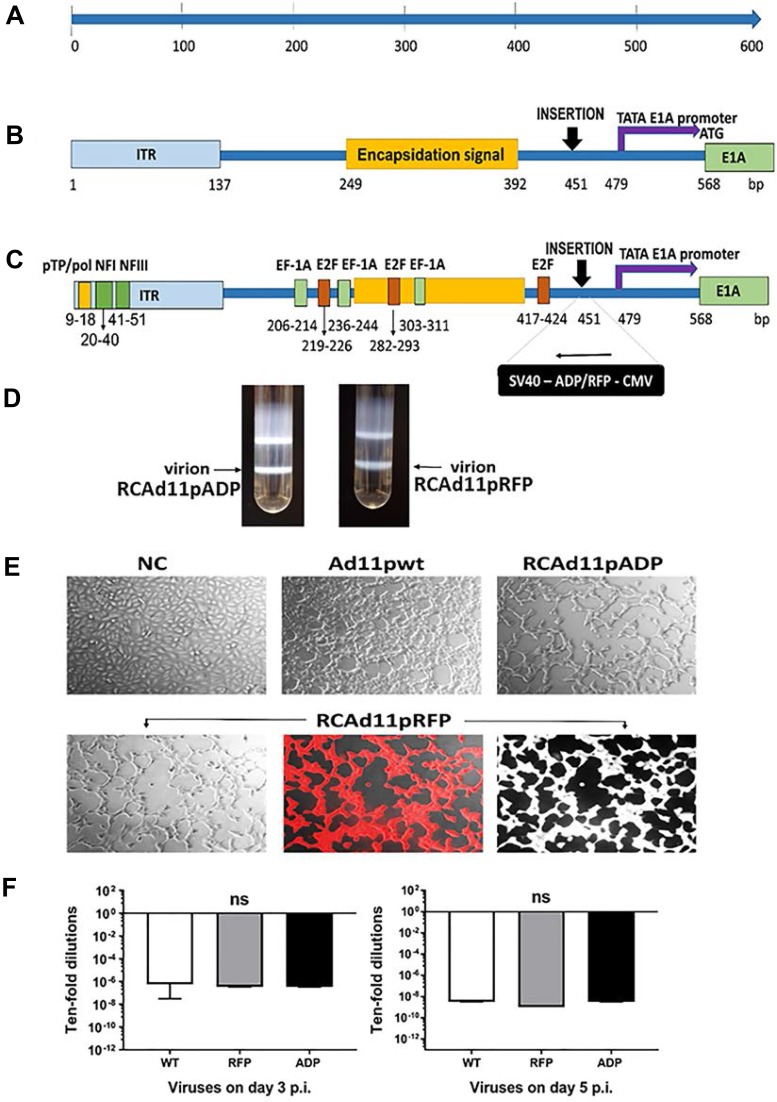
Schematic representation of the adenovirus 11p E1A promoter (nt 1-568) and the insertion sites in the RCAd11pADP and RCAd11pRFP vectors (**A**) The left end of the Ad11p genome (nt 1-568). (**B**) The distal region upstream of the E1A promoter, the left end of the Ad11p genome covering the left ITR and the encapsidation signal region, TATA box and E1A promoter. (**C**) Important motifs: the distal region indicated with a yellow box shows the conserved AT-rich sequence that is key for binding of the pTP/pol complex. The predicted sequence location for nuclear factor (NF) I and III are marked with a dark green box in the ITR region. The sequences corresponding to EF-1A and E2F, transcription factor-binding sites, are indicated with a light green box and an orange box, respectively. ADP and RFP expression cassettes were introduced 451 bp upstream of the Ad11p genome in the reverse orientation. ADP: adenovirus death protein. RFP: red fluorescence protein. CMV: cytomegalovirus. SV40: simian virus 40 polyadenylation sequence. The TATA box and E1A promoter are marked with a purple arrow and start at nt 479. The start codon (ATG) of the E1A ORF is indicated at nt 568. (**D**) RCAd11pADP and RCAd11pRFP were purified by CsCl ultracentrifugation. The arrow indicates the virion band. (**E**) Viral infectivity was monitored by microscopy. A549 cells were infected with Ad11pwt, RCAd11pRFP, and RCAd11pADP at 360 VPs/cell (equal to 0.1 pg/cell) or PBS as a control. At 24 h p.i., cells infected with all three viruses were monitored by light microscopy and red fluorescence microscopy to detect RCAd11pRFP. Images were obtained at 200X magnification. (**F**) Viral infectivity at 3 and 5 days p.i. was determined through a TCID_50_ assay using the Reed-Muench accumulative method. Data are shown as averages ± SEs from four samples; ns indicates that the differences between viruses were not significant (*P* > 0.05).

The strategy used to construct recombinant replication-competent adenovirus 11p ADP (RCAd11pADP or ADP) and RFP (RCAd11pRFP, or RFP) upstream of the E1 region in the Ad11p genome is illustrated in Figure 1A–1C. The genome sizes of the adenoviruses were as follows: RCAd11pADP, 36,105 bp, corresponding to an increase of 3.77% compared with the unmodified Ad11pwt genome (34,794 bp); RCAd11pRFP, 36,504 bp, corresponding to an increase of 4.91% compared with the Ad11p genome. The CsCl density of the two vectors was comparable (Figure 1D). The infectivity of each vector used in this study was measured based on A549 cell CPE at 24 h p.i. (Figure 1E), and that of the three viruses was further determined through TCID_50_ measurements. No significance difference in viral titres was found between 3 and 5 days p.i., as shown in Figure 1F. Moreover, RCAd11pRFP and RCAd11pADP were at least as potent as the Ad11pwt virus in A549 cells. Thus, RCAd11pADP and RCAd11pRFP display the same full replication capacity as Ad11pWT and can also express the ADP and RFP genes, respectively, as shown in Figure 1A–1F.

### RCAd11p vectors demonstrated high levels of replication in PC3 and DU145 cells in a qPCR assay

**Figure 2:**
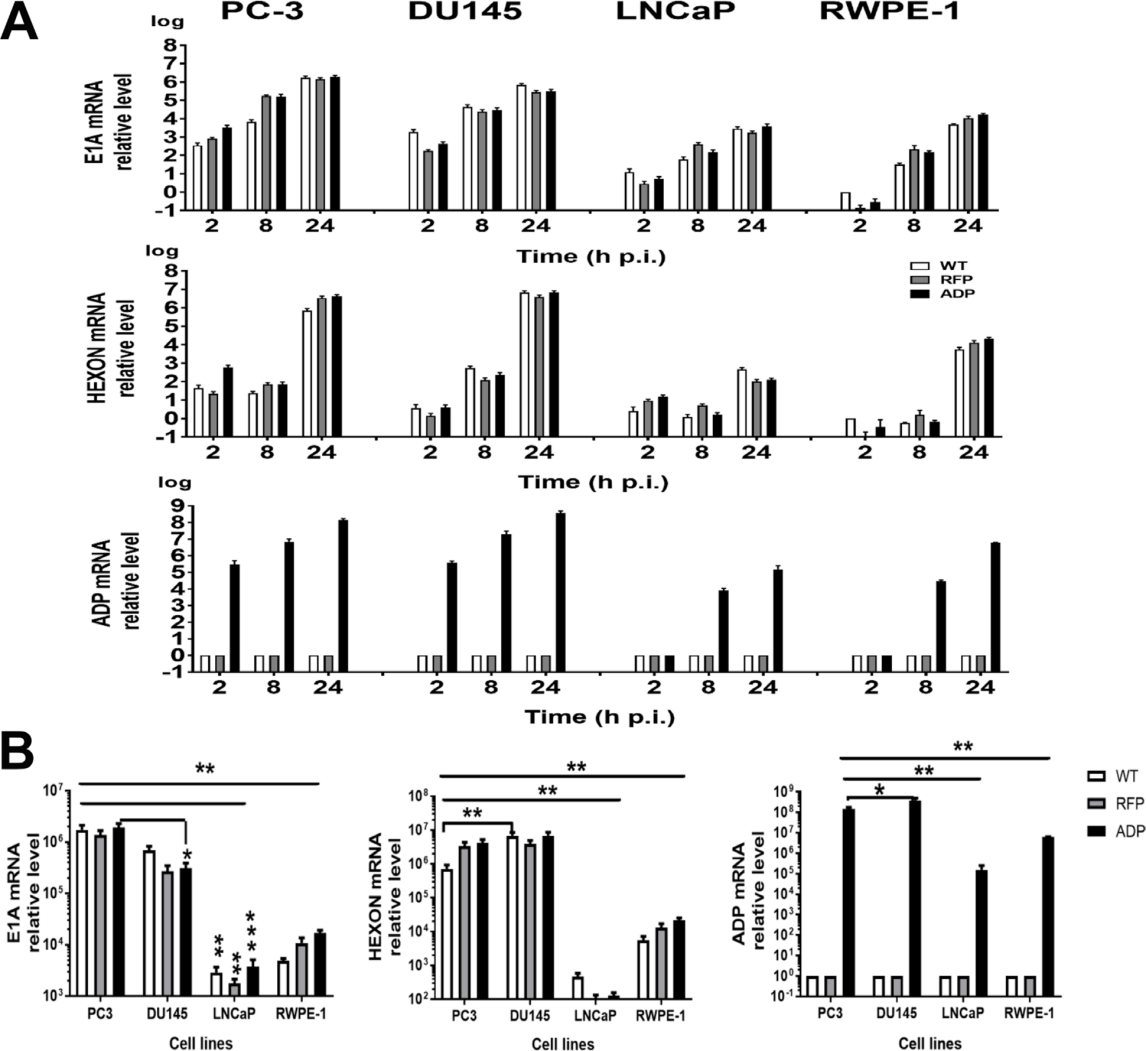
Quantification of virus and vector replication in prostate carcinoma cells PC3, DU145, LNCaP and RWPE-1 cells were infected with 360 VPs/cell of the Ad11pwt RCAd11pADP, and RCAd11pRFP viruses (VPs/cell). The cells were harvested at 2, 8, and 24 h p.i.; total DNA was extracted, and viral DNA was quantified by qPCR. The raw qPCR data were normalized to the β-actin expression level in each test. The statistical significance (*P* value) of differences among the viruses and cell lines at the three time points was assessed. (**A**) qPCR for E1A, hexon, and ADP gene expression. (**B**) Statistical analysis of E1A, hexon, and ADP gene expression in all four cell lines at 24 h p.i. The asterisks indicate a significant difference, as determined by an unpaired *T* test ^*^P < 0.05; ^**^P < 0.01; ^***^P < 0.001. The data are shown as averages ±SEs from three samples.

We measured E1A, hexon, and ADP mRNA levels by quantitative real-time PCR after reverse transcription to determine whether the amounts of proteins measured by antibody staining correlated with the mRNA levels. Total RNA from PC3, DU145, LNCaP, and RWPE-1 cells infected with the RCAd11pADP, RCAd11pRFP and Ad11pwt viruses was isolated at 2, 8, and 24 h p.i. The mRNA levels of each detected gene were quantified using primers targeting the coding regions of E1A, hexon and ADP and normalized with respect to the initial mRNA levels at 2 h p.i. As shown in Figure 2A, E1A mRNA levels were increased at 2 h p.i. and reached a peak at 24 h p.i., indicating that E1A expression increased in a time-dependent manner. PC3 and DU145 cells contained significantly higher quantities of E1A mRNA than did LNCaP and RWPE-1 cells. The time point at which hexon mRNA expression was detected was several hours later than that at which E1A expression was observed, beginning to be detected at 8 h p.i. and increasing to high levels at 24 h p.i. In agreement with the E1A expression findings, PC3 and DU145 cells also demonstrated higher hexon mRNA expression compared with LNCaP cells at the same time point. ADP mRNA was only expressed in the cells after infection with the RCAd11pADP virus, with marked variability in level. At 2 h p.i, ADP was not detected in LNCaP and RWPE-1 cells but was detectable in PC3 and DU145 cells (Figure 2A). At subsequent time points, ADP mRNA levels in PC3 and DU145 cells were significantly higher than those in LNCaP and RWPE-1 cells, resulting in 2–3 log higher expression in PC3 and DU145 cells compared with LNCaP and RWPE-1 cells. The differences between PC3 cells and LNCaP or RWPE-1 cells infected with each virus were statistically significant, as indicated in Figure 2B. Thus, the results of the RT-PCR assay indicate that viral replication was dependent on the tumour cells targeted.

### E1A, hexon and ADP expression levels showed marked variability in the cell lines studied

**Figure 3:**
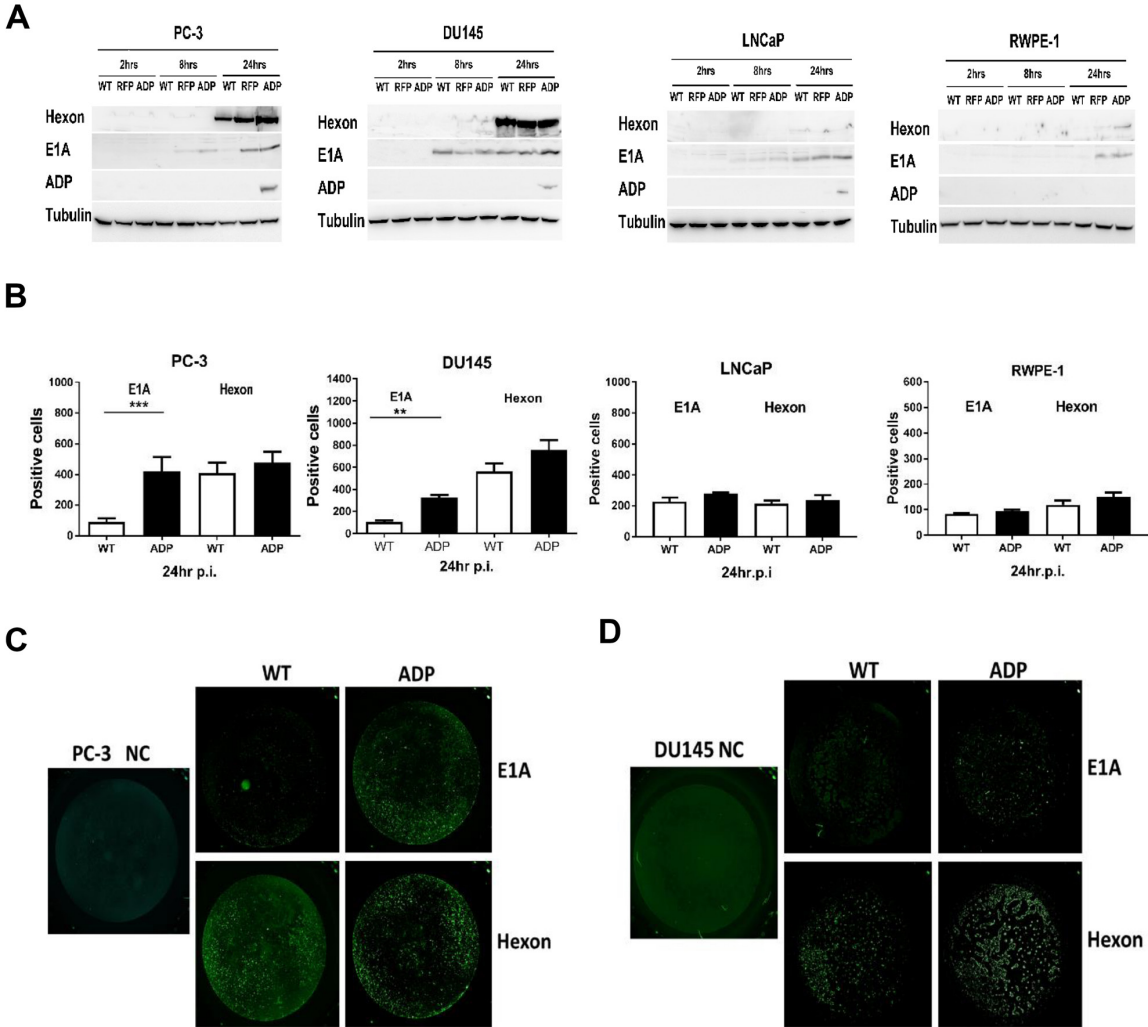
Western blotting and Trophos assays for detecting E1A, hexon, and ADP protein expression (**A**) E1A, hexon, and ADP protein expression in PC3, DU145, LNCaP and RWPE-1 cells infected with viruses was assayed by Western blotting at 2, 8, and 24 h p.i. The data shown are representative of three independent experiments. Tubulin was used as a loading control. (**B**–**D**) PC3 and DU145 cells showed high E1A and hexon protein expression, as demonstrated by a Trophos assay. Cells were infected with the Ad11pwt and RCAd11pADP vectors at 3600 VPs/cell (IP/VP≥1:72); after incubation for 24 h, the cells were stained with a rabbit anti-Ad11p E1A or Ad11 virion antibody and then with swine anti-rabbit IgG-FITC. The fluorescent signal was quantified using a Trophos plate runner. The graphs show relative gene expression levels compared with that obtained with the control Ad11pwt. Significant differences in E1A protein and hexon expression were observed. The asterisks indicate a significant difference, as demonstrated by an unpaired *T* test (^*^P < 0.05; ^**^P < 0.01; ^***^P < 0.001); ns, not significant; au, arbitrary units. The data are shown as the averages ± SEs from three to five samples.

A Western blotting assay was performed to obtain a better understanding of viral protein expression. At 24 h p.i., higher E1A protein expression was detected in cells infected with the RCAd11pRFP and RCAd11pADP viruses than in cells infected with the Ad11pwt virus (Figure 3A). Additionally, hexon was efficiently expressed in PC3 and DU145 cells, and its expression was generally more stable in cells after infection with the ADP virus than in those infected with the WT virus. ADP protein was detectable in PC3, DU145 and LNCaP cells at 24 h p.i. but could hardly be found in RPWE-1 cells. Based on the mRNA and protein levels measured, Ad11p viruses are more strongly expressed in highly aggressive cancer cells than in normal cells (Figure 3A).

### E1A expression is enhanced in PC3 and DU145 cells infected with ADP vectors

To quantify E1A and hexon expression in prostate cancer cells and normal prostate cells infected with RCAd11pADP and Ad11pwt viruses, polyclonal antibodies against the E1A peptide or hexon in Ad11p viruses were used as primary antibodies, and polyclonal antibodies against rabbit IgG conjugated to FITC were employed as secondary antibodies. Because the wavelengths of conjugated FITC and the RFP protein overlap slightly, we were barely able to distinguish between the fluorescence intensities of FITC and RFP, resulting in our inability to detect hexon protein expression mediated by the RFP vector in the Trophos assay.

The total numbers of positive cells in 96-well plates were calculated, and each cell line infected with RCAd11pADP and Ad11pwt was compared. E1A expression in PC3 and DU145 cells was notably enhanced 4.0- and 3.0-fold after infection with the ADP virus (Figure 3B–3D), respectively. In contrast, LNCaP and RWPE-1 cells displayed only slightly increased E1A gene expression, and enhanced E1A protein expression was observed only with those vectors in which the expression cassette was inserted in the upstream E1 region. The E1A protein of oncolytic adenovirus vectors is a known tumour killer. In this study, hexon expression levels were also evaluated, and the cell lines exhibited limited viability after WT and ADP virus treatment. In general, hexon expression was higher in PC3 and DU145 cells than in LNCaP and RWPE-1 cells. The vector with the E1 insertion resulted in enhanced E1A gene expression. Comparison of the ADP vector and Ad11pwt virus indicated that hexon expression was relatively stable. In summary, E1A expression in PC3 and DU145 cells was mediated by RCAd11pADP but not notably enhanced by the Ad11pwt virus, as demonstrated by the Trophos assay.

### RCAd11pADP exerts the highest CPE

**Figure 4:**
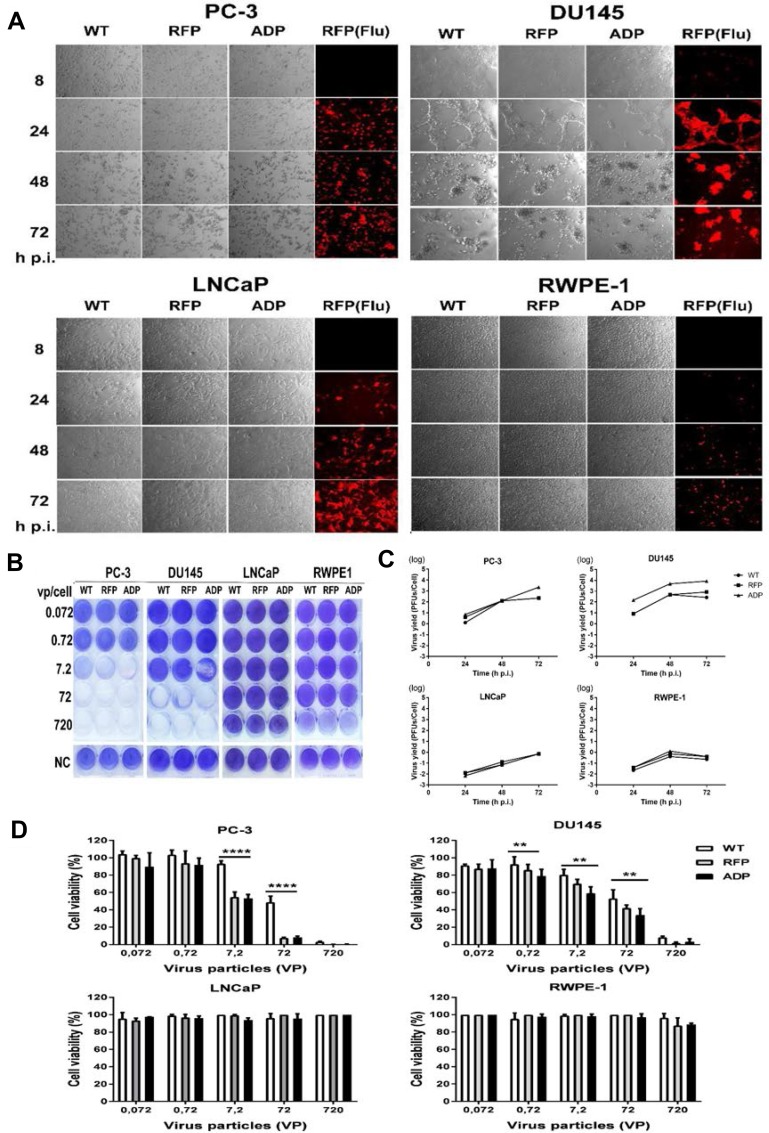
(**A**) Transduction and CPE assay based on red fluorescence microscopy and phase-contrast light microscopy. PC3, DU145, LNCaP and RPWE-1 cells were transduced with RCAd11pADP/RFP and Ad11pwt at 360 VPs/cell. At 8, 24, 48 and 72 h p.i., cells expressing RFP and cells exhibiting CPE were detected by fluorescence microscopy and light microscopy (200X magnification). (**B**) Toxicity assay. RCAd11pADP resulted in enhanced oncolytic activity in metastatic prostate cells. ADP, RFP, and WT viruses at the indicated dosages were incubated with the following prostate cell lines: PC3, DU145, LNCaP, and RPWE-1 cells. At 6 days p.i., the cells were fixed and stained with crystal violet. Three independent experiments were performed, and representative results are shown. (**C**) Production of ADP, RFP and WT infectious particles in PC3, DU145, LNCaP, and RPWE-1 cells. Cultures of 10^5^ PC3, DU145, LNCaP and RPWE-1 cells were infected with Ad11pwt or two RCAd11p vectors at 720 VPs/cell and incubated for 24, 48, and 72 h. The infected cells were then freeze-thawed three times, and endpoint titrations of the lysates were performed in A549 cells to determine the production of infectious particles. The results are presented as the means ± 95% confidence intervals from at least four independent experiments. (**D**) Cell viability was measured using a CellTiter-Glo^®^ luminescent cell viability kit at 6 days p.i. The graph presents the averages from triplicate samples. The asterisks indicate significant differences, as demonstrated by an unpaired *T* test (^*^P < 0.05; ^**^P < 0.01; ^***^P < 0.001); ns, not significant. The data are presented as the average ± SEs from three samples.

To investigate the efficacy of Ad11p infection in prostate cancer cells, four cell lines were infected with the three viruses. CPE was detected in PC3 and DU145 cells at 24 h p.i., and a very strong effect was observed at 72 h p.i. In contrast, LNCaP cells infected with these viruses did not clearly demonstrate a CPE at the same time points. The normal control line, RPWE-1 cells, displayed limited RFP expression but an undetectable CPE (Figure 4A). Overall, the viral infection data reveal that the Ad11pwt, ADP and RFP vectors exhibit tumour-specific replication activity in prostate cancer cells.

### The RCAd11pADP vector induces efficient cytolysis in PC3 and DU145 cells

We subsequently employed an *in vitro* cytotoxicity assay to investigate whether ADP overexpression mediated by the RCAd11p vector is able to increase oncolytic activity. Various cell lines (PC3, DU145, LNCaP, and RWPE-1) were infected with tenfold dilutions of Ad11pwt, RCAd11pRFP, and RCAd11pADP. At a high dilution of virus particles (7.2 VPs/cell), RCAd11pADP led to the complete destruction of PC3 cells and 50% destruction of DU145 monolayers at 6 days p.i. As expected, PC3 and DU145 cells infected with RCAd11pRFP remained 20% and 90% viable, respectively. In contrast, cells infected with the Ad11pwt virus remained 60% more viable in PC3 cells or 100% more viable in DU145 cells at the same time point. No obvious viral destruction was observed for monolayers of LNCaP and RWPE-1 cells, as shown in Figure 4B.

### Ad11p viruses produce the highest virus titres in PC3 and DU145 cells

Our investigation of the production of infectious virions in prostate cancer cell lines revealed that PC3 and DU145 cells were permissive to Ad11p virus and vector infection but that LNCaP and RPWE-1 cells were refractory. We then determined whether the rate of viral particle production was high in PC3 and DU145 cells, as suggested by structural protein expression, and whether any infectious particles were produced in refractory LNCaP and RPWE-1 cells. Cultures of PC3, DU145, LNCaP, and RPWE-1 cells individually infected with Ad11pwt, RCAd11pRFP and RCAd11pADP at 720 VPs of VPs/cell were incubated for 24, 48, and 72 h; the cells were freeze-thawed, and the lysates were titrated in A549 cells. The RCAd11pADP titres in PC3 and DU145 cells were increased by 2-3 logs at 48 and 72 h, respectively.

As shown in Figure 3C, the titres of the three viruses in PC3 and DU145 cells were higher than those in LNCaP and RWPE-1 cells. Moreover, at 72 h p.i., RCAd11pADP produced a 1-log greater amount of virus in PC3 cells compared with Ad11pwt and RCAd11pRFP. At the three tested time points, the virus yield of RCAd11pADP in DU145 cells was higher than those of Ad11pwt and RCAd11pRFP by approximately 1 log of magnitude. In contrast, Ad11pwt and RCAd11pADP/RFP replication appeared to be restricted in LNCaP and RWPE-1 cells. Nonetheless, no distinct differences in replication were observed among the three viruses in LNCaP and RWPE-1 cells (Figure 4C).

### RCAd11pADP significantly reduces proliferation in PC3 and DU145 cells but not in LNCaP and RWPE-1 cells

To investigate the effect of the RCAd11p vectors and Ad11pwt virus on cell viability and proliferation, the three prostate metastatic cell lines were infected with the viruses. As shown in Figure 4D, the viability and proliferation of PC3 and DU145 cells were markedly reduced after infection with 7.2, 72, and 720 VPs/cell, respectively. A significant difference was found between RCAd11pADP and RCAd11pRFP on one side and Ad11pwt on the other side. PC3 and DU145 cells were more sensitive than were LNCaP and RWPE-1 cells. The strongest viability was observed with 720 VPs/cell, which caused the death of more than 95% of PC3 cells and 90% of DU145 cells, but with limited or no effect on LNCaP and RWPE-1 cells, at 6 days p.i.

### RCAd11pADP shows improved anti-tumour effects *in vivo* experiments and prolongs mouse survival

We further evaluated the tumour-killing efficacy of RCAd11pADP through two *in vivo* animal experiments. In the first experiment, nude mice were sacrificed on the same day, and the tumour size and weight were measured. In the second experiment, nude mice were observed over an extended period to determine survival rates. The tumours grew at a markedly faster rate in the untreated control group (PBS) than in the groups treated with Ad11pwt and the RCAd11pADP/RFP vectors. The mean tumour size and weight in the Ad11pwt group were reduced to 40% of the corresponding values in the mock group (PBS). Indeed, the tumour size in the Ad11pwt group was twofold greater than that in the RCAd11pADP group. Thus, RCAd11pADP exerts predominant anti-tumour effects and displays increased efficiency compared to the Ad11pwt virus in Balb/c nude mice. No side effects were observed after virus injection.

**Figure 5:**
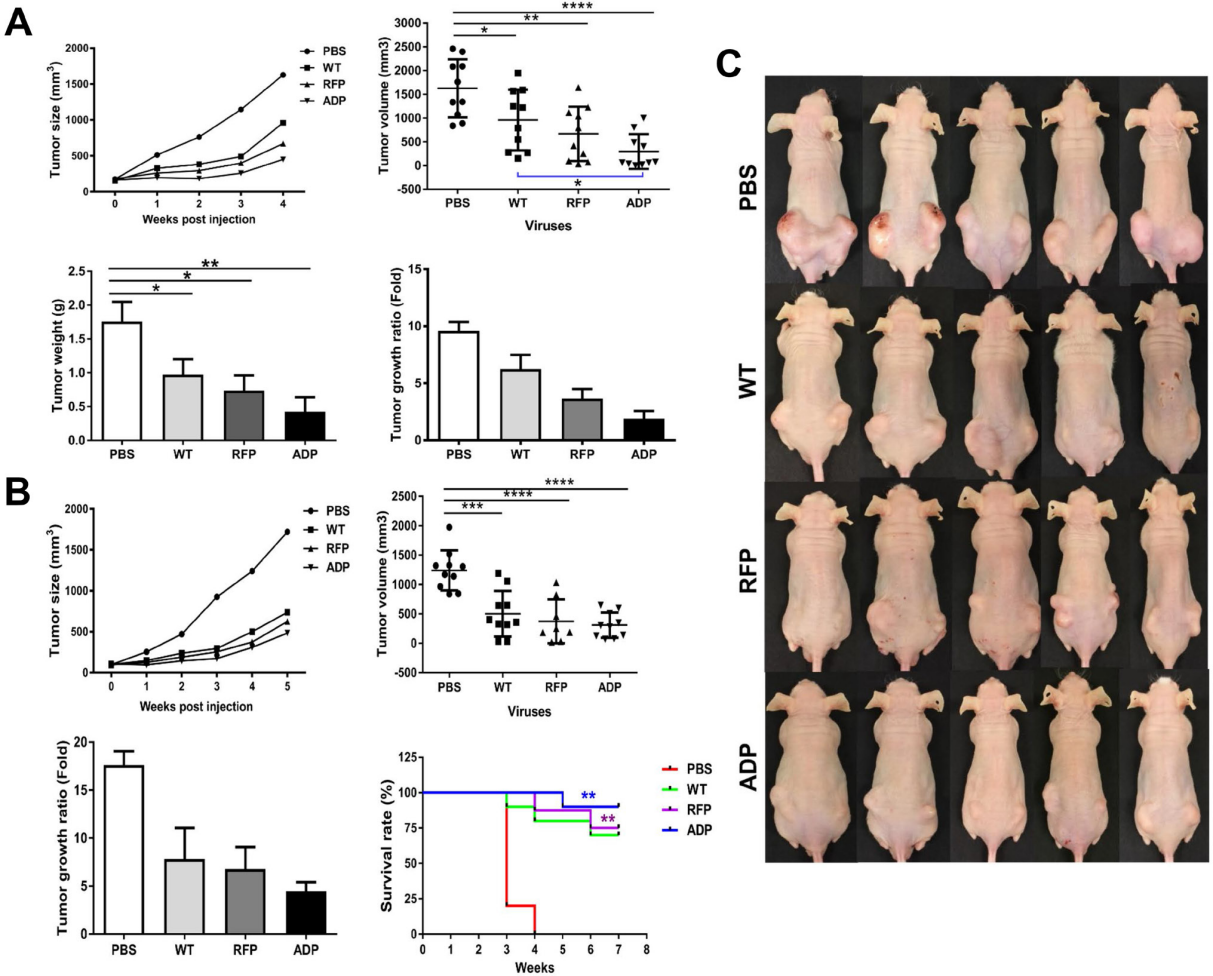
Anti-tumour effects of RCAd11pADP, RCAd11pRFP and Ad11pwt viruses on PC3 tumours in BALB/c mice An *in vivo* oncolytic model with RCAd11pADP and Ad11pwt was established. Three million PC3 cells were subcutaneously transplanted into the left and right flanks of Balb/c nude mice. The PC3 tumours grew to at least 75 mm^3^ by 2.5 weeks p.i. Subcutaneous tumours derived from human prostate cancer PC3 cells were injected intra-tumourally with 10 μg (equal to 3.5 × 10^10^ VPs) of Ad11pwt, RCAd11pRFP and RCAd11pADP per tumour; the mice in the control group were injected with PBS. After one week, a secondary virus injection was performed with 3.57 × 10^10^ VPs/tumour, and the tumour volume was recorded weekly. An *in vivo* oncolytic model with RCAd11pRFP and Ad11pwt was also established, and the mean tumour volume and weight were summarized according to the virus treatment. Survival rates with and without Ad11pwt, RCAd11pRFP and RCAd11pADP treatment were also monitored. A summary of animal experiments I and II are shown in (**A**, **B**) respectively. The oncolytic effects mediated by Ad11pwt, RCAd11pRFP, and RCAd11pADP on PC3 tumours in xenograft mice were evaluated. The data represent the means and standard errors of the mean (SEM) from 10 tumours from five mice at 4 weeks after intra-tumour injection of Ad11pwt, RCAd11pRFP, and RCAd11pADP viruses. ^*^P < 0.05; ^**^P < 0.01; ^***^P < 0.001; ^****^P < 0.0001). Survival analysis (**B** (**bottom right**)): Percent survival was determined by monitoring death (tumour size > 850 mm3) over a period of 7 weeks. Mice treated with RCAd11pADP exhibited a significant survival advantage over those treated with PBS. The statistical significance of the survival differences was tested by the log-rank (Mantel-Cox) test. The bars indicate the mean values, and the asterisks indicate statistical significance (^*^P < 0.05, ^**^P < 0.01). In the survival experiment, the mouse was sacrificed if one side of tumour was larger (> 850 mm/tumour) than the other side, according to ethical permission. (**C**) Tumour morphology observed in animal experiment II at 4 weeks after intra-tumour injection of PBS or WT, RFP or ADP viruses.

In general, the tumour inhibition results obtained in the second animal experiment agree with the results from the first experiment. RCAd11pADP enhanced tumour suppression compared with that observed in the WT and RFP groups, though the survival rates of the virus-treated groups differed from that of the PBS-treated group (Figure 5A–5C). A higher number of mice in the ADP vector-treated group survived compared with the groups treated with RFP or WT virus. The tumour morphologies before and after viral treatment in each group were imaged before viral treatment and over 4 weeks p.i., respectively (Figure 5A–5C).

### RCAd11pADP exerts notably enhanced apoptotic effects

**Figure 6:**
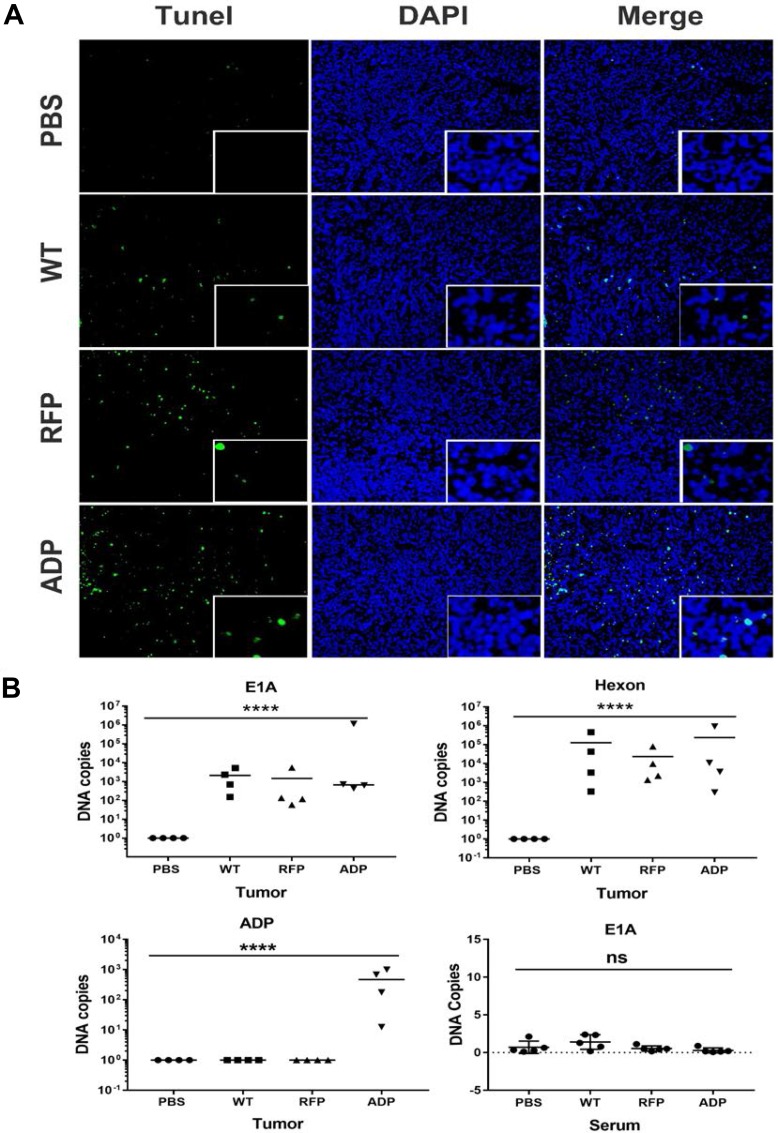
(**A**) Detection of apoptosis in PC3 xenografts. At the end of the experiment, apoptosis was detected in tumour sections using a TUNEL assay. We rarely observed apoptotic fluorescent nuclei in PBS-treated tumour tissues, whereas tumours infected with Ad11pwt or RCAd11pRFP exhibited limited numbers of apoptotic fluorescent nuclei. In contrast, tumours treated with RCAd11pADP contained large numbers of apoptotic fluorescent nuclei. Adenovirus 11p DNA in samples of prostate tumours and sera from Balb/c nude mice was detected by qPCR. The data were obtained from four tumours per group and analysed in triplicate. (**B**) Four weeks after intra-tumour injection, the expression levels of the E1A and hexon genes of Ad11p and the ADP gene in samples of PC3 tumours and blood were detected by qPCR. Exploratory qPCR analysis of PC3 xenograft mice provides supporting evidence demonstrating that Ad11pwt, RCAd11pRFP and RCAd11pADP can replicate in tumour mass for a long period of time and inhibit tumour growth. A bar indicates the mean values for a group. Significance was assessed by one-way ANOVA with Bartlett's test. (^*^P < 0.05; ^**^P < 0.01; ^***^P < 0.001; ^****^P < 0.0001). The data are presented as the averages ± SEs from three samples.

Tissue sections on slides were subjected to TUNEL assays, and tumours infected with the RCAd11pADP virus showed the highest degree of apoptotic cells, as shown in Figure 6. As apoptosis is an important cause of tumour suppression, immunofluorescence imaging for detecting apoptosis was performed to examine the function of RCAd11pADP as a tumour suppressor in PC3 cells. A higher rate of apoptosis and greater numbers of fluorescent cells were observed in these cells compared with the PBS, Ad11pwt, and RCAd11pRFP groups, as revealed by microscopy. The results of the apoptosis assay in tumour cells were consistent with earlier reports that ADP induces cell killing via both caspase-dependent and caspase-independent mechanisms [15]. Based on these results, we hypothesize that RCAd11pADP accelerates the progression of apoptosis in PC3 cells.

### Ad11p virus or vector DNA was detectable in tumour masses at 4 weeks p.i. but not in mouse sera

To explore the apoptotic effect of RCAd11p vectors, the level of Ad11p DNA four weeks after intra-tumour administration was detected by qPCR. E1A, hexon and ADP gene expression in four tumour samples from each group was quantified, and the results demonstrated that E1A and hexon were detected in all analysed tumour samples after virus treatment but not in the mock control group (PBS treatment). Furthermore, the ADP gene was only detected in the RCAd11pADP-treated group. Different DNA copy numbers obtained in the qPCR assay were also detected, likely due to local delivery of the virus to the tumour mass, which can result in the collection of tumour tissue from an area with a low viral distribution. However, in general, the E1A gene copy numbers obtained for Ad11pwt, RCAd11pRFP, or RCAd11pADP in tissues were similar, with values of approximately 10^2^−10^3^ copies. In contrast, relatively high hexon gene expression was found in each tumour tissue sample, with values of approximately 10^3^−10^6^ copies. Neither E1A nor hexon expression was detected in serum samples. As the detection of E1A and hexon in tumour samples by qPCR supports the results of the apoptotic assay, the exploratory qPCR analysis of PC3 xenograft mice provides evidence demonstrating that Ad11pwt, RCAd11pRFP and RCAd11pADP can replicate in a tumour mass for a long period of time and inhibit tumour growth.

### RCAd11pADP caused significant apoptosis and cell death

**Figure 7:**
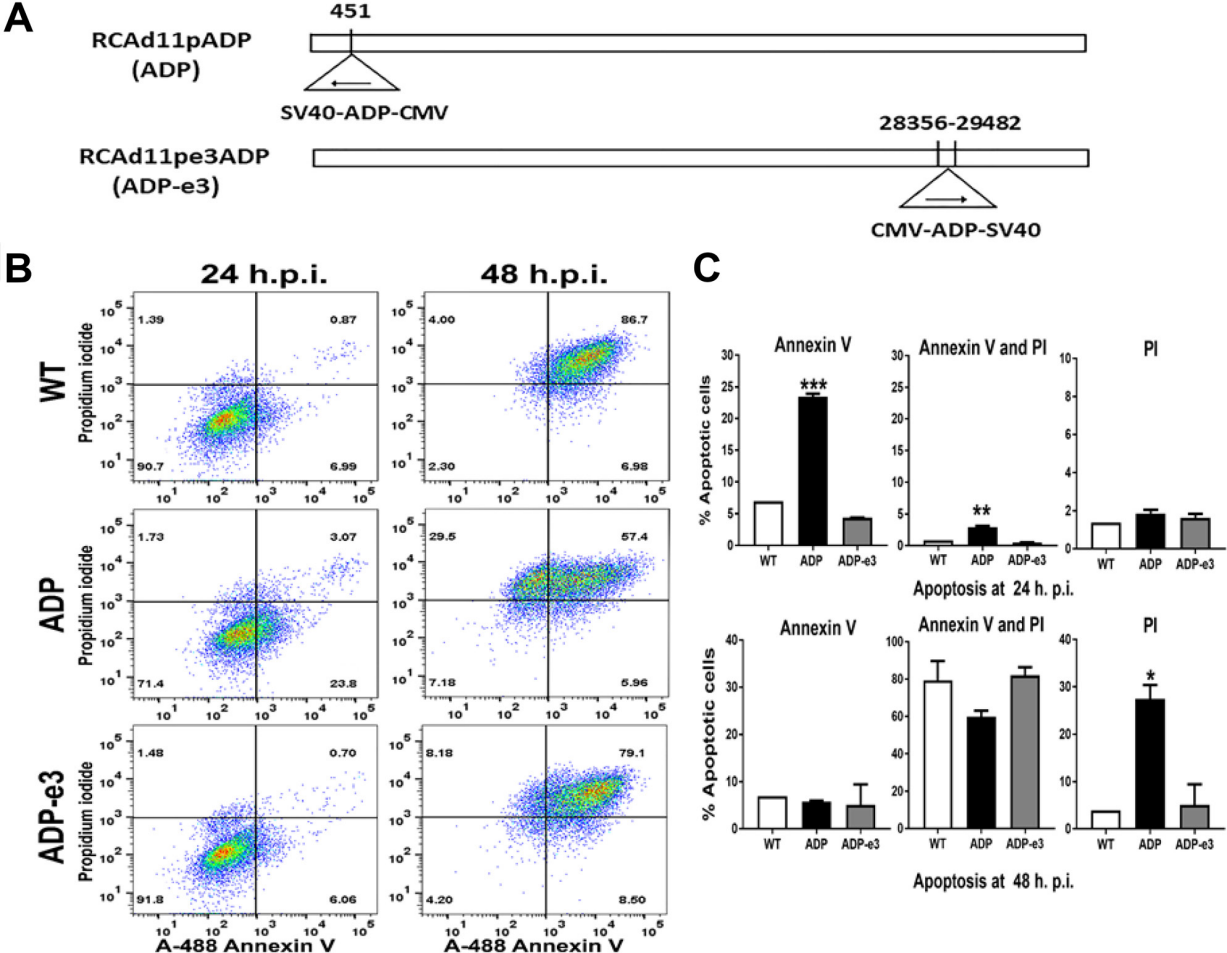
The RCAd11pADP virus induced significant apoptosis compared to that induced by RCAd11pE3ADP and Ad11pwt virus in PC3 cells (**A**) Two replicating adenovirus 11p vector constructs: ADP insertion at early region 1 or 3 of the adenovirus 11p genome. (**B**) PC3 cells were infected with 720 VPs/cell of WT, ADP, and ADP-e3 viruses. After 24 or 48 h of incubation, the cells were analysed by flow cytometry, and phosphatidylserine translocation to the outer membrane was measured by Annexin V activity. Dead cells were assessed by flow cytometry with 488 and 575 nm excitation sources. Virus-induced early apoptosis (%) is summarized in the lower right quadrant, marked with Annexin V. For apoptosis (%), as indicated in upper right quadrant, cells were double positive for Annexin V and PI. Late apoptotic cells (%), as summarized in the upper left quadrant, were stained only with PI, with most cells being dead. (**C**) Statistical analysis for early apoptosis, apoptosis, and late apoptosis induced by the indicated viruses. The ADP virus induced a more rapid apoptotic effect than did ADP-e3 or Ad11pwt at 24 hr. p.i.; statistical significance was assessed by one-way ANOVA, with a *P* value < 0.001. Similarly, more double staining was found for PC3 cells transfected with ADP than for cells transfected with the ADP-e3 and WT viruses; *P* value < 0.01. Regarding late apoptosis, the ADP virus versus ADP-e3 and WT was statistically significant (*P* value < 0.05). The data shown are representative of at least two experiments using triplicates for each sample.

PC3 cells were infected with RCAd11pADP at a concentration of 720 VPs/cell; after 24 h, 23.8% of cells were positive for Annexin V in the lower right quadrant, indicating early apoptosis (Figure 7B). With the same quantity of Ad11pwt or ADP-e3 viral infection, only 6.99% and 6.06% of cells were positive for Annexin V, respectively. Moreover, ADP resulted in 3.07% of cells being double stained (Annexin V and PI), in the upper right quadrant in Figure 7B. In contrast, only 0.87% and 0.70% of cells infected with Ad11pwt and ADP-e3 stained double positive. After increasing the infection period to 48 h, the proportion of PI-positive cells increased to 29.9% of dead cells infected by ADP, whereas Ad11pwt and ADP-e3 infection resulted in 4% and 8.0% PI positivity in the upper left quadrant, indicating that WT and ADP-e3 caused much less apoptosis than did ADP. Interestingly, double the degree of late apoptosis was found for ADP-e3 compared with Ad11pwt at 48 h p.i., indicating the ADP-e3 has a role in PC3 cell apoptosis (Figure 7B). Regarding the statistical analysis, ADP induced significant early apoptosis (*P* < 0.001) with Annexin V-positive cells and apoptosis-positive cells with both Annexin and PI in comparison to Ad11pwt and ADP-e3 at 24 h p.i. (*P* < 0.01, Figure 7C). At 48 h p.i., the ADP virus triggered significant cell death compared to WT and ADP-e3 (statistical significance: *P* < 0.05, Figure 7C). These results indicate that ADP induced markedly enhanced apoptosis compared to Ad11pwt and ADP-e3, whereas ADP-e3 triggered apoptosis somewhat later than did ADP, with insertion in the upstream E1 region.

## DISCUSSION

In the present study, we compared fully replication-competent adenovirus 11p expressing the ADP gene from the upstream E1 region with the Ad11pwt or RFP vector for use in prostate cancer therapy. We investigated the transduction, protein expression, viral replication and oncolytic efficacy of the novel Ad11p vectors and demonstrated that the oncolytic activities of the ADP vector include tumour-specific cell killing and that this effect is dependent on ADP and E1A gene expression as well as viral replication ability in targeted cells. Moreover, the novel oncolytic adenoviral 11p ADP vector significantly enhanced inhibition of metastatic prostate activity *in vitro* and *in vivo*.

The first aspect we addressed is the selectivity of the three Ad11p virus vectors for metastatic prostate suppression. We examined the E1A transcription patterns of the vectors in four prostate cell lines. *In vitro* experiments showed that the Ad11pwt virus or RCAd11p vectors generally resulted in 2-3-log higher mRNA levels in PC3 and DU145 cells than in LNCaP and RPWE-1 cells. Regardless of whether the ADP gene was incorporated, the Ad11pwt virus caused no detectable cytolytic effects in LNCaP or RWPE-1 cells. Our results indicate the occurrence of tumour-specific replication in highly metastatic cells. The RCAd11p vectors possess a native genetic feature that restricts their replication in cancerous cells, and the viruses replicated much more poorly in normal or low-malignancy prostate cells. A previous study indicated that a cellular protein that activates transcription factors stimulates the transcription of multiple E1A-inducible early promoters [17], and it was later demonstrated that the E2 promoter of the adenoviral genome possesses a cellular factor reorganization domain that is recognized by a cellular component termed E2F [18, 19]. The complex consisting of the E2 promoter and E2F activates E1A gene transcription. As E2F is likely an oncogene with markedly variable activity, the E1A gene exhibits greater activity in tumour cells than in normal cells. The E2 promoter in Ad11p variants contains a sequence domain recognizing a cellular factor in PC3 and DU145 cells, facilitating complex formation and resulting in the activation of E1A gene transcription. In LNCaP and RWPE-1 cells, however, the cellular factor/oncogene does not trigger E1A gene transcription, which limits or renders E1A transcription undetectable and thereby results in non-lytic infection. Thus, various cellular factors induce differential cytolytic effects in prostate cell lines.

Because ADP affects cell viability, we compared expression of ADP, a representative early gene, and hexon, a representative late gene, in the cell lines with detectable E1A gene transcription. ADP expression in cells infected with RCAd11pADP and/or RCAd11pRFP, as well as the Ad11pwt virus, was evaluated by antibody staining. At 24 h p.i., detectable hexon expression and a strong CPE was found in 90% of PC3 and DU145 cells. Furthermore, the oncolytic effects of the RCAd11pADP and RCAd11pRFP vectors were 2-5-fold more efficient than were those of the Ad11pwt virus in PC3 or DU145 cells (Figure 4). Western blotting and Trophos assays revealed the E1A gene to be highly expressed in PC3 and DU145 cells infected with RCAd11pADP and RCAd11pRFP. Although quantification of the hexon structural protein did not reveal notable differences between the virus and vectors, obviously increased E1A protein expression was detected with the RCAd11pADP virus, suggesting that the observed oncolytic effects were partially mediated by the function of E1A. E1A of adenoviruses plays an important role in binding to or stimulating other cellular factors, either through virus replication or by activating cellular pathways [20–22]. In human cells, E1A expression does not result in malignant transformation but rather elicits an anti-tumour effect in many cancer types, and it is thus considered a tumour suppressor. E1A-mediated inhibition of tumour development involves reduction of HER2/neu expression as well as mechanisms unrelated to HER-2. E1A negatively regulates cellular proteins that are important for gene transcription, such as NF-kB, and reduces tumour metastasis and promotes apoptosis induced by serum deprivation. Thus, the rate of viral DNA replication and the virus yield are comparable. Importantly, the E1A gene was overexpressed with the RCAd11pRFP and RCAd11pADP vectors containing an inserted foreign gene expression cassette in the upstream E1A promoter compared with the Ad11pwt virus.

Lysis and death of cells infected with lytic viruses are accelerated by ADP [23], which facilitates the efficient release of progeny virions without affecting the yield of the progeny virus. Because the impact of ADP on virus yield from infected prostate cells has not been previously described, we compared virus yields from four cell lines infected with Ad11pwt or ADP/RFP-expressing viruses. Although yields differed among the cell types investigated in this study, we found that ADP expression had no impact on the total yield of progeny virus from a given cell type. Accordingly, an Ad5wt virus carrying a native ADP in its E3 region did not exhibit a high rate of viral propagation, as observed in previous studies and by our experimental data (unpublished). Conversely, an Ad41 vector carrying the ADP gene in its E3 region increased both cell proliferation and yield [24].

The tumour-killing efficacy of RCAd11pADP became obvious when the vectors were tested in a more rigorous model in which the vectors were administered intra-tumourally for the treatment of subcutaneous PC3 tumours. In this system, only a fraction of the injected vector dose reached the target tumours; thus, the few infectious viral particles must replicate sufficiently to exert an effect on tumour growth. RCAd11pADP and RCAd11pRFP affected prostate tumours much more efficiently than did Ad11pwt (Figure 5). The mean tumour volume following treatment with the ADP vectors was less than 1/4 of the untreated tumour volume or half of the tumour size following Ad11pwt treatment. Thus, among the three viruses tested in the animal model, RCAd11pADP can be considered to be a largely effective oncolytic virus. This finding is particularly interesting because it might reflect effects on overexpression of both ADP and E1A observed in our *in vitro* experiments.

In these *in vivo* experiments, we reduced the viral dose to 10 μg/tumour, and each tumour was injected twice with this dose. In our previous study [13], we used one injection of a dose of 50 μg/tumour. Thus, the RCAd11pADP viral dose was reduced to only 40% of that used in our previous study [12, 13]. As the RCAd11pADP vector was more sensitive and efficient than was Ad11pwt, the prostate tumours treated with the ADP vector were more efficiently suppressed, and animal survival was prolonged by at least three weeks compared with that of the untreated animals.

Several studies have investigated whether substitution of the E1A promoter with other tumour-specific promoters can enhance anti-tumour specificity. However, there are few studies to date that have considered the importance of maintaining the cis-acting element and TATA box for adenoviral replication. Fortunately, due to the biology of the RCAd11p vectors, the inserted expression cassette is transcribed and translated with the therapeutic or marker gene. The cis-acting element and TATA box enhance expression of the downstream E1A gene, and the E1A trans-acting element also stimulates the ADP expression cassette. Thus, the cis-trans-acting elements exert a substantial effect on E1A and ADP gene expression [25, 26].

We observed increased E1A gene expression in our *in vitro* experiments and showed that both the ADP and RFP vectors suppressed tumour growth and promoted apoptosis *in vivo* to a greater extent than did the Ad11pwt virus. E1A-mediated apoptosis was also observed *in vivo*, suggesting that E1A activity contributes to tumour suppression. ADP protein overexpression induces cell killing via both caspase-dependent and caspase-independent mechanisms [15]. Therefore, as demonstrated in the present study, the prostate tumour suppressor mediated E1A and ADP gene expression. In the Ad11p vector construct, the expression cassette is placed upstream of the cis-acting element and E1A gene. This cis-acting sequence plays a key role in activating viral transcription, and when it is deleted or mutated, viral replication is notably reduced by at least tenfold [25, 27]. Therefore, a replication-competent adenovirus vector should retain the cis-trans sequence to preserve the native replication capacity of the adenovirus. Although we demonstrated that the novel Ad11p vector with an insertion of 1400–1750 nt in front of the cis-activating region functions well, we must also establish the maximal capacity of the insertion site in the upstream E1A region and determine the insertion size that inactivates cis-trans activity. Ad11pwt, a parental virus for replication-competent adenovirus vectors, does not cause clinically significant illness in immunocompetent adults and is rapidly eliminated by the immune system. We expect that a fully replication-competent adenovirus vector might exhibit similar characteristics in patients. The ADP gene caused increased oncolytic effects in metastatic tumour cell lines but did not induce a clear CPE in normal cells in this study. We obtained similar amounts of virus particles from the cells infected with RCAd11pADP compared with from those infected with the parental virus Ad11pwt. Therefore, our findings suggest that ADP-mediated tumour suppression blocks multiple pathways in cancer cells, resulting in cell death. It was previously reported that Ad11 with a modified Ad5 E1 fragment more broadly targets tumour cells than does the Ad11 genome [28]. Although Ad11 evokes immediate inflammation, similar to Ad5, repeated administration of Ad11 is better tolerated in mice compared to Ad5; long-term fibrotic tissue remodelling is also reduced in mice treated with Ad11 [29]. In our animal experiments, potentially strong side effects after virus injection were not detected. In addition, the RCAd11pADP virus particles did show higher efficacy at lower doses. Accordingly, this vector might offer a therapeutic window comparable to or even wider than that of a tumour-restricted vector. Furthermore, unlike many tumour-restricted adenovirus vectors, RCAd11pADP possesses WT replication capability and is easy to produce at the high titres required for clinical applications. Such vectors might also be injected intra-tumourally, an approach that largely limits viral dissemination to injected local cells.

The Ad11p virus offers several advantages within the context of improved delivery to tumours, including the use of CD46 and desmoglein-2 as primary receptors, rapid release from a cell, and lower seroprevalence. Ad11p vectors target haematopoietic cells. Although several research groups recently reported Ad11-based vectors, RCAd11p vector constructs have marked differences. In general, Ad11 exhibits enticing potential as a virotherapy agent [30]. Accordingly, a variant of Ad11 (ColoAd1) was recently investigated in phase I clinical trials for the treatment of solid tumours [31]. Adenovirus replication generally induces lysis in host cells. It has been reported that species C adenovirus kills host cells via apoptotic machinery-independent necrosis-like death [15]. It was later found that Ad5 causes cell lysis through autophagy and autophagy-triggered caspase activity, but investigations of cell lysis induced by Ad11 virus are limited. A recent study conducted by Dyer *et al*. [1] demonstrated that one of species B adenoviruses, Enadenotucirev, mediates non-apoptotic cell death through membrane disruption and the release of inflammatory mediators [1]. In the present study, our RCAd11pADP vector induced E1A and ADP expression in PC3 cells, and these two genes triggered apoptosis in our *in vivo* experiments. Interestingly, Ad55, a recombinant adenovirus strain derived from Ad11 and Ad14, induces milder apoptosis in infected cells [32]. RCAd11pADP triggered significantly enhanced apoptosis in comparison to Ad11pwt or RCAd11pE3ADP. Disruption of the apoptotic pathway is a major factor in the multistep tumourigenesis process, whereas the induction of apoptosis mediated by our RCAd11pADP vector will be important for tumour suppression in pre-clinical and clinical trials.

In summary, the purpose of these studies was to evaluate the oncolytic toxicity effects of the RCA11pADP virus in a preclinical model with the aim of providing a scientific basis for its application in humans. We found that RCAd11pADP enhances anti-tumour effects on metastatic prostate cells. ADP might not only induce the release of a greater number of viral particles but may also affect the E1A gene, which accelerates the virus lifecycle. Our findings suggest that both the ADP gene and E1A gene play key roles in tumour suppression and apoptosis during prostate metastasis *in vitro* and *in vivo*.

## MATERIALS AND METHODS

### Ethics statement

This investigation was conducted in accordance with the ethical standards and national and international guidelines and was approved by the Umeå Animal Research Ethics Committee (registration number, A26-14; applicant, Ya-Fang Mei).

### Replication-competent adenovirus 11p vectors

The ADP gene, which is located from 29,491 to 29,772 nt in the Ad5 E3 region, was cloned into the Ad11pe1 shuttle vector (Ad5 genome accession no. M73260, version M73260.1). The gene encoding red fluorescence protein (RFP) was obtained from the pVisionRFP-N plasmid (https://www.biovision.com, Bio Vision Research Products, CA, USA). An RCAd11p vector carrying ADP or RFP under the control of the CMV promoter and the SV40 polyA sequence was constructed by inserting the gene into the Ad11p E1 shuttle vector at 451 nt upstream of the E1A region [11]. A linear shuttle vector carrying the ADP/RFP-expressing cassette as well as the full-length Ad11p genome was obtained by homologous recombination in BJ5183 Escherichia *coli* cells. The recombinant plasmid of the ADP/RFP-Ad11p genome was propagated in DH5α cells, and purified pAd11pADP/RFP was digested with the NotI restriction enzyme and transfected into A549/293 cell lines using Lipofectamine^TM^ 2000 (Invitrogen) for harvesting of infectious particles. To evaluate the infectivity of the vectors, a monolayer of A549 cells was infected at a tenfold dilution, and the cytopathic effect (CPE) was monitored daily. TCID_50_ was calculated 3 and 5 days post-infection (p.i.).

To elucidate functional differences with ADP insertion at the E1 or E3 region of the adenovirus 11p genome, we also introduced a DNA fragment of CMV-ADP-SV40 into the E3 shuttle vector at 28356-29482 nt of the Ad11p genome [11]. The two arms of the shuttle vector were released by NaeI digestion and then cloned into the Ad11p genome through homologous recombination in BJ5183 cells [11]. pAd11pE3ADP can release the Ad11p genome expressing ADP from the E3 region by NotI digestion. The linearized Ad11p genome carrying the ADP cassette was transfected into A549 cells using Lipofectamine 2000 (Invitrogen). CPE was detectable after 4–7 days p.i., and the transfected cells were harvested and kept at −80°C as viral stocks. The ADP expression cassette in RCAd11pe3ADP was obtained by restriction enzyme digestion with KpnI and HindIII.

### Cell lines and cell culture

A549 cells, a human line derived from an oat cell carcinoma in the lung, was selected to produce human adenovirus serotype 11 virus, a prototype of Ad11 (Ad11p). The cells were grown in DMEM supplemented with 5% foetal bovine serum (FBS) (HyClone). Three prostate cancer cell lines were also used: PC3, derived from a metastatic bone site, and LNCaP, derived from a metastatic lymph node site, were grown in DMEM containing 10% FBS (HyClone); DU145, derived from a metastatic brain site, was grown in RPMI-1640 containing 10% FBS (HyClone). A normal cell line, RWPE-1, is of human epithelial origin; RWPE-1 cells were grown in keratinocyte serum-free medium (K-SFM) supplemented with 0.05 mg/mL BPE and 5 ng/mL EGF [33]. All cells were cultivated in a 5% CO_2_ atmosphere at 37°C. During viral infection, the FBS concentration was reduced to 50% of that in the growth medium for all cell lines except RWPE-1.

### Virus preparation and measurement of viral concentrations

All viruses were propagated in A549 cells. Virion particles were purified using CsCl gradients as described previously [34]. The bands were collected, and the virion concentrations were determined by spectrophotometry at 260 and 330 nm according to the following formula: 1 OD of absorbance at A260-A330 was assumed to be equivalent to 10^12^ viral particles (VPs)/mL or 280 μg/mL. Viral quality was evaluated by determining TCID_50_ in A549 cells as the number of infectious virus particles (IPs) versus the total VPs. In detail, 1.5 × 10^4^ A549 cells were seeded in each well of a 96-well plate and cultivated overnight; the cells were then inoculated with tenfold-diluted viruses from 7.2 × 10^4^ VPs/well to 7.2 × 10^−2^ VPs/well. CPE was monitored daily. Batches of purified virus with an IP:VP ratio of at least 1:72 were considered acceptable for use in experiments.

### Antibodies

To evaluate the expression levels of Ad11p E1A proteins and ADP protein, we generated polyclonal rabbit antibodies against the Ad11p E1A peptide and anti-ADP peptide. Specifically, the adenovirus 11p E1A ORF contains three ORFs that share limited homology [35]. The E1A ORFs of Ad11p share a common amino acid sequence at the N terminus, (NH2)-MRDLRFLPQEIISAETGNEC-(COOH), from amino acid (AA) 1 to AA 19. The ADP gene in the Ad5 E3 region consists of 93 AAs. An antibody against ADP was prepared using the (NH_2_)-CHPNNDGIHRLDGLKH-(amidated) peptide with a peptide encompassing AAs 72–86 of the ADP gene. Rabbit anti-E1A peptide polyclonal antibodies and rabbit anti-ADP peptide polyclonal antibodies were produced by Agrisera (Sweden). Further purification was conducted using an affinity peptide column. Rabbit polyclonal antibodies and a β-tubulin loading control were purchased from Abcam (ab6046). The secondary antibody, horseradish peroxidase-conjugated goat anti-rabbit IgG, was obtained from Santa Cruz Biotechnology (sc-2030). Blots were developed using Pierce™ Fast Western Blot Kit with the ECL substrate according to the manufacturer's instructions and imaged using a Fujifilm LAS 4000 (GE Healthcare Life Sciences).

### Adenovirus infection of prostate cancer cells

Cells were seeded in 25-cm^2^ flasks and infected the next day with one of the three vectors at 720 VPs/per cell at 37°C with shaking for 1 h. The medium was then replaced with medium containing 2% FBS, and the vectors were incubated for 8, 24, 48 or 72 h p.i. Images were captured at the indicated time points using an Axiovert 25-Carl Zeiss microscope. The adenovirus yield from each cell line after incubation for 24, 48, and 72 h p.i was determined as described above.

The cells and media were collected and freeze-thawed three times, and virus titration was performed using A549 cells with the limiting dilution method. Four replicates were used for each sample, and viral titres were recorded daily.

### Cytotoxicity assay

To evaluate the oncolytic effects of adenovirus on prostate cancer cells, cells in 24-well plates were infected with tenfold dilutions of the Ad11pwt, RCAd11pRFP and RCAd11pADP vectors at 720, 72, 7.2, 0.72, and 0.072 VPs/cell and incubated at 37°C for 6 days p.i. Cytotoxicity was assessed by crystal violet staining. Briefly, the cell medium was removed, and the cells were fixed in 4% paraformaldehyde (PFA) at room temperature for 3 min. The plates were washed with phosphate-buffered saline (PBS) and stained with 1% crystal violet in 70% ethanol for 3 min. After staining, the cells were rinsed three times with water and air dried for imaging [12].

### Cell proliferation assay

To evaluate the oncolytic effects of the three viruses in prostate cancer cells, cells were infected with tenfold dilutions of RCAd11pADP, RCAd11pRFP and Ad11pwt from 7.2 × 10^2^ to 7.2 × 10^−2^. Cell viability was determined using the CellTiter-Glo^®^ luminescent cell viability kit at 6 days p.i. (cat. G7571, Promega). Quantification was performed using a plate reader (Tecan Infinite F200 Pro).

### qPCR for E1A, hexon and ADP genes

Sample of 1.5 × 10^6^ cells were infected with Ad11pwt, RCAd11pRFP, or RCAd11pADP at 360 VPs/cell; after the virus was allowed to absorb at 37°C for 1 h, the cells were washed twice with DMEM. DMEM containing 2% FCS was then added, and the cells were incubated for 2, 8, and 24 h p.i. Total cellular RNA was isolated using the TRIzol LS reagent (Life Technologies), treated with DNase I and converted to cDNA using Moloney Murine Leukaemia Reverse Transcriptase (M-MLV RT) reagents (Invitrogen). Quantitative PCR was performed using SYBR-Green Master Mix (Life Technologies) with a Step One Plus™ real-time PCR system (Applied Biosystems). The following primers were employed to detect Ad11p E1A and the hexon gene: qPCR E1A-Forward primer, 5′-GTCCTGTGTCTGATGCTGATGAAT3′; and qPCR E1A-Reverse primer, 5′-ACAGGAATGG GCTTGCGCAC-3′. Briefly, quantitative real-time PCR was performed using a degenerate primer pair, Kadgen1 (forward)-Kadgen2 (reverse) (5′-CWTACA TGCACATCKCSGG-3′ and 5′-CRCGGGCRAAYTGC ACCAG-3, respectively; DNA Technology A/S, Aarhus, Denmark), specific for the conserved region of the Ad11p hexon gene and detects all human adenoviruses [11]. For quantitative analysis, the purified viral genome from each virus or vector was assessed by spectrophotometry (NanoDrop, ND-1000 Spectrophotometer, Live Science Saveen Werner, Sweden; http://www.swab.se) and diluted tenfold to generate a standard curve. The raw qPCR data were normalized according to the level of β-actin expression in each sample and calculated as mRNA copies.

### Immunoblot analysis

Cells were harvested at 24 h p.i. and lysed in buffer (50 mmol/L HEPES, pH 7.4, 250 mmol/L NaCl, 1 mmol/L EDTA, 1 mmol/L DTT, 1 mmol/L NaF, and 1% Triton X-100) containing protease inhibitors. Total protein was separated on a 12% NuPAGE^®^ Novex Bis-Tris gel under reducing conditions and transferred to a polyvinylidene fluoride membrane (Millipore). The membrane was blocked with Blotto/Tween (5% non-fat dry milk, 0.2% Tween 20, in PBS) and probed with the following primary antibodies: rabbit anti-hexon polyclonal antibody (KS590 from Virus Lab), rabbit anti-E1A peptide antiserum polyclonal antibody, rabbit anti-ADP peptide polyclonal antibody, and rabbit anti-tubulin polyclonal antibody (Abcam, no. ab6046). The secondary antibody was horseradish peroxidase-conjugated goat anti-rabbit IgG (Santa Cruz Biotechnology, no. SC-2030). The blots were developed using ECL substrates (Pierce™ Fast Western Blot kit) and imaged according to the manufacturer's instructions using a Fujifilm LAS 4000 (GE Healthcare Life Sciences).

### Immunodetection of E1A and hexon gene expression

E1A and hexon expression in the four cell lines after infection with the Ad11pwt, RCAd11pRFP and RCAd11pADP vectors was measured as follows. Cells (1.5 × 10^4^) were seeded per well in a 96-well plate and incubated overnight at 37°C. The next day, the cells were infected with each virus at 3600 VPs/cell. At 24 h p.i., the cells were centrifuged at 300 × g for 5 min, and the supernatants were removed by aspiration. The cells were then washed once with 200 μL of PBS and fixed with 100 μL of 3% PFA in PBS for 30 min at RT. The cells were labelled using a rabbit anti-Ad11p E1A peptide antibody at 1:500 dilution or Ad11 virion antibody (KS590, virus lab at UmU) at 1:1,000 dilution in PBS. For antibody labelling, the cells were incubated with the antibody while shaking at 4°C for 1 h and then washed three times with PBS. The cells were visualized using polyclonal swine anti-rabbit immunoglobulins/FITC (Dako Denmark A/S, cat. no. F0205) at 1:200 dilution in PBS and DAPI at 1:5,000 dilution for 30 min at 4°C. The cells were then washed three times with PBS, and fluorescence was measured using a Trophos plate reader.

### Tumour xenograft experiments

Balb/c nude mice at 4 weeks of age were purchased from Scanbur A/B (Silovej 16–18, DK-2690 Karlslunde, Denmark; https://www.scanbur.com) and quarantined for 1 week prior to initiation into the study. The animal experiments were conducted in accordance with the Guide for the Care and Use of Animals for research purposes and were approved by the Committee of Animal Ethics at Umeå University (license no. A26-14). PC3 cells (3 × 10^6^) were injected subcutaneously into the posterior flanks of the mice. Mice bearing tumours with a volume of 100-150 mm^3^ (2-3 weeks after PC3 cell inoculation) were randomly assigned into four groups (*n* = 5). The tumours were injected with a single dose of 3.57 × 10^10^ VPs or 10 μg of adenovirus in 50 μL of PBS, and the mice in the control group were injected with 50 μL of PBS. The intra-tumoural viral injections were repeated one week later. The volumes of the implanted tumours were measured weekly using a Vernier calliper and calculated by the following formula: tumour volume (mm^3^) = ½ (length × width × height). Four weeks after the first adenovirus injection, the mice were sacrificed, and the tumours were weighed. Half of each tumour was processed for immunohistochemistry analysis, such as an apoptotic assay, whereas the other half was immediately frozen in liquid nitrogen and processed for viral genome analysis or replication. The surviving proportion of mice was recorded weekly according to tumour size.

### qPCR of tumour tissues for viral genes

A 20-mg sample of prostate tumour tissue in 20 μL of Proteinase K plus 400 μL of DNA pretreatment buffer was shaken for 20 s in a homogenizing instrument, and 200 μL of the sample was transferred to a new tube for robotic DNA preparation (Arrow NorDiag, DiaSorin AB). The DNA from tumour cells was eluted in 50 μL of elution buffer and assessed at 260/280. The highest DNA concentration was 250–500 ng/μL. A 1-μL aliquot of the DNA sample was used for qPCR. DNA from mouse serum was also prepared for qPCR; 200 μL of serum was prepared for total DNA using a QIAamp DNA blood mini kit (Qiagen) and eluted in 50 μL of elution buffer. The protocols used for the qPCR assay and calculation were the same as those used for cell line qPCR.

### Apoptosis assay

Tumours were isolated, fixed in 4% PFA in PBS for at least 24 h, and embedded in paraffin. Five-micron-thick sections were cut and mounted, and apoptosis in the tumours was confirmed by *in situ* fluorescence terminal deoxynucleotidyl transferase-mediated dUTP-fluorescein nick end labelling (TUNEL), according to the manufacturer's recommended protocol (Roche, Switzerland). The slides were treated with 50 μL of 4,6-diamidino-2-phenylindole solution (5 μg/mL) for 10 min to stain the nuclei and then washed three times. Images were obtained using a Leica microscope.

### Annexin V *in vitro* assay

PC3 cells (10^6^) were infected with 720 VPs/cell of Ad11pwt, RCAd11pADP (ADP gene inserted at the E1 region) and RCAd11pe3ADP. After 1 h incubation at 37°C with slow shaking, the cells were washed once with DMEM medium and then continually cultivated with 2% FBS in DMEM at 37°C for 24 or 48 hours. Preparation of a negative control was carried out by incubating cells in the absence of viruses. Cell were detached with 0.5% EDTA-PBS, washed once with cold PBS and stained with Alexa Fluor 488 according to the manufacturer's protocol (Cat no. V13245, Invitrogen). The stained cells were analysed by flow cytometry (BD LSR II), with fluorescence measurement at 575 nm emission and 488 nm excitation.

### Statistical analysis

Statistical analyses (*t* tests and one-way ANOVA) were performed with GraphPad Prism software version 7.0 (GraphPad Software, San Diego, CA, USA).

## Abbreviations

RCAd11p: replication-competent adenovirus 11p vector
ADP: adenovirus death protein
RFP: red fluorescence protein
CAR: coxsackie-adenovirus receptor
Ad11pwt: adenovirus 11 prototype wild type
RCAd11pRFP/RFP: replication-competent adenovirus 11p vector expressing RFP from upstream early region 1
RCAd11pADP/ADP: replication-competent adenovirus 11p vector expressing ADP from upstream early region 1
RCAd11pe3ADP/ADP-e3: replication-competent adenovirus 11p vector expressing ADP from early region 3
UmU: Umeå University.
